# The effect of SGLT-2i administration on red blood cell distribution width in patients with heart failure and type 2 diabetes mellitus: A randomized study

**DOI:** 10.3389/fcvm.2022.984092

**Published:** 2022-09-29

**Authors:** Nikolaos Katsiadas, Andrew Xanthopoulos, Grigorios Giamouzis, Spyridon Skoularigkis, Niki Skopeliti, Evgenia Moustaferi, Ioannis Ioannidis, Sotirios Patsilinakos, Filippos Triposkiadis, John Skoularigis

**Affiliations:** ^1^Department of Cardiology, Konstantopoulio General Hospital, Nea Ionia, Greece; ^2^Department of Cardiology, University Hospital of Larissa, Larissa, Greece; ^3^Hematology Laboratory, Konstantopoulio General Hospital, Nea Ionia, Greece; ^4^1st Department of Internal Medicine, Diabetes Center, Konstantopoulio General Hospital, Nea Ionia, Greece

**Keywords:** sodium glucose co-transporter-2 inhibitors, red blood cell distribution width, mechanisms, aging, oxidative stress, erythropoietin

## Abstract

**Background:**

Recent studies suggest that the pivotal mechanism of sodium glucose co-transporter-2 inhibitors (SGLT-2i) favorable action in patients with heart failure (HF) and type 2 diabetes mellitus (DM) is the stimulation of erythropoiesis *via* an early increase in erythropoietin (EPO) production which leads to hematocrit rise. Red blood cell distribution width (RDW) is a simple hematological parameter which reflects the heterogeneity of the red blood cell size (anisocytosis). Since, EPO has been also implicated in the pathophysiology of RDW increase, the current mechanistic study examined the effect of SGLT-2i administration on red blood cells size (RDW) in patients with HF and DM.

**Methods:**

The present was a prospective single-center study. Patients (N=110) were randomly assigned to dapagliflozin (10 mg a day on top of antidiabetic treatment) or the control group. Inclusion criteria were: (a) age > 18 years, (b) history of type 2 DM and hospitalization for HF exacerbation within 6 months. The evaluation of patients (at baseline, 6 and 12 months) included clinical assessment, laboratory blood tests, and echocardiography. Data were modeled using mixed linear models with dependent variable the RDW index. In order to find factors independently associated with prognosis (1-year death or HF rehospitalization), multiple logistic regression was conducted with death or HF rehospitalization as dependent variable.

**Results:**

An RDW increase both after 6 and after 12 months was observed in the SGLT-2i (dapagliflozin) group (*p* < 0.001 for all time comparisons), whereas RDW didn't change significantly in the control group. The increase in RDW was positively correlated with EPO, while negatively correlated with ferritin and folic acid (*p* < 0.005 for all). Baseline RDW was significantly associated with 1-year death or rehospitalization, after adjusting for group (SGLT-2i vs. control), age, gender, smoking and BMI at baseline.

**Conclusion:**

RDW increased with time in patients with HF and DM who received SGLT-2i (dapagliflozin). The increased RDW rates in these patients may stem from the induction of hemopoiesis from dapagliflozin. Baseline RDW was found to be independently associated with outcome in patients with HF and DM.

## Introduction

Red blood cell distribution width is a simple parameter of the complete blood count (CBC) which reflects the heterogeneity of the red blood cell size (anisocytosis) and has been traditionally used for the classification of several types of anemia (1, 2). Over the last decade, high RDW values have been associated with adverse outcomes in patients with cardiovascular disease including stable coronary artery disease, acute coronary syndromes, stroke, diabetes mellitus (DM), and heart failure (HF) (3–7). DM is a major risk factor for new-onset HF and vice versa (8). An increase in Hemoglobin A1C (HbA1C) of 1% correlates to an increment of 8% in HF, whereas diabetic patients have an almost twofold increased risk of HF (9, 10). Interestingly, it has been reported that red blood cell distribution width (RDW) is a good prognostic marker in patients with HF and DM (11). Reduced iron mobilization, oxidative stress, renal failure as well as ineffective erythropoiesis, have been implicated in the pathophysiology of RDW increase (12). However, the exact pathophysiological mechanism of RDW increase in HF, DM, and other pathological states, remain unclear (11).

Sodium glucose co-transporter-2 inhibitors (SGLT-2i) are a new class of antidiabetic drugs which reduce hyperglycemia through inhibition of glucose reabsorption in the renal proximal tubules (8). SGLT-2i administration has been associated with favorable cardiovascular outcomes (13, 14). Although several hypotheses have been proposed (including diuresis and natriuresis, reduction in blood pressure and afterload, direct effects on myocardial sodium and calcium handling, alterations in myocardial energetics, and improved progenitor cell response), recent studies in patients with type 2 DM suggest that the pivotal mechanism of SGLT-2i favorable action is the stimulation of erythropoiesis *via* an early increase in erythropoietin (EPO) production which leads to hematocrit (Ht) rise (15, 16). Since erythropoietin has been implicated in the pathophysiology of RDW increase as well, the current mechanistic study examined the effect of SGLT-2i administration on red blood cells size (RDW) in patients with HF and DM.

## Methods

### Study patients

The present was a prospective single-center study which took place during the period from 4-2020 to 7-2021 on the Cardiology Department of Konstantopoulio General Hospital (Greece). Patients were randomly assigned *(Random allocation software)* (17) to dapagliflozin (10 mg a day on top of antidiabetic treatment) or the control group (no change in antidiabetic treatment) (1:1) in an open-label fashion. Inclusion criteria were: (a) age > 18 years, (b) history of type 2 DM and hospitalization for HF exacerbation within 6 months. Exclusion criteria were: (a) current or prior treatment with SGLT-2i or gucagon-like peptide 1 (GLP1) agonists, (b) blood transfusions or ferrum or folic acid or vitamin B12 administration the last 6 months, (c) glomerular filtration rate (GFR) < 30 mL/min/1.73 m^2^, (d) active cancer, or (e) predicted survival <1-year. NK and EM generated the random allocation sequence, enrolled participants, and assigned participants to the study groups.

The evaluation of patients (at baseline, 6 and 12 months) included clinical assessment, laboratory blood tests, and echocardiography. Levels of Ht and RDW were measured with the use of the Unicel DxH 600, Beckman USA analyzer on samples obtained for standard of care evaluation. The normal reference range for age, sex, and ethnicity using NHANES III normal range data for RDW in the hospital laboratory was 11.5–15% with intra-assay variation of 2.6% and inter-assay variation of 1.5% (18). Ferrum, ferritin, vitamin B12, folic acid and EPO measured with the use of Access 2, Beckman USA analyzer, while HbA1C, glucose, urea, creatinine, electrolytes, serum glutamic-oxaloacetic transaminase (SGOT), serum glutamic pyruvic transaminase (SGPT), brain natriuretic peptide (BNP), uric acid, and troponin with Dimension EXL, Siemens analyzer. Echocardiography was performed and reviewed by two independent echocardiographers, with the use of GE, HealthcareVivid e95.

This study conforms to the principles outlined in the Declaration of Helsinki and was approved by the institutional review committee. All patients provided written their informed consent.

### Definitions

Diabetes mellitus was defined as HbA1C ≥ 6.5% and history of antidiabetic treatment (19–22). Heart failure was defined as a clinical syndrome consisting of cardinal symptoms (e.g., breathlessness, ankle swelling, and fatigue) that may be accompanied by signs (e.g., elevated jugular venous pressure, pulmonary crackles, and peripheral oedema). It is due to a structural and/or functional abnormality of the heart that results in elevated intracardiac pressures and/or inadequate cardiac output at rest and/or during exercise (23, 24).

### Outcomes and follow up

The aim of this study was to compare the RDW longitudinal changes between the group of patients who received SGLT-2i (dapagliflozin) and the control group. Furthermore, we examined the association between RDW changes with time and clinical parameters that were known from the literature to be associated with RDW. Lastly, we investigated whether baseline RDW was independently associated with the combined endpoint of death or HF rehospitalization at 12 months. The study follow up was 1-year.

### Statistical analysis

Quantitative variables were expressed as mean (Standard Deviation) or as median (interquantile range). Qualitative variables were expressed as absolute and relative frequencies. For the comparison of proportions chi-square tests were used. Independent samples Student's *t*-tests and Mann-Whitney tests were used for the comparison of quantitative variables between the two groups. Wilcoxon test was used for the time comparisons of RDW, in each group separately. Data were modeled using mixed linear models with dependent variable the RDW index. Primarily, the regression equation included terms for time, group, the interaction term of time and group as well as all characteristics that differed significantly between the two groups at baseline (Model 1). Secondly, another model was constructed with terms for time, group, the interaction term of time and group as well as all characteristics that were known from literature to be associated with RDW (Model 2). Adjusted regression coefficients (β) with standard errors (SE) were computed from the results of the mixed models. Hypothesized interactions of variables in the models were checked. Log transformations were used in RDW due to lack of normal distribution. In order to find factors independently associated with prognosis, multiple logistic regression was conducted with 1-year death or HF rehospitalization as dependent variable. As independent variables RDW (at baseline), group (SGLT-2i vs. control), gender, age, smoking and body mass index (BMI) (at baseline) were used. Adjusted odds ratios (OR) with 95% confidence intervals (95% CI) were computed from the results of the logistic regression analyses. ROC curves (Receiver operating characteristic curves) were used in order to estimate the prognostic value of RDW regarding poor prognosis (1-year death or HF rehospitalization), for each group separately. Sensitivity and specificity were calculated for optimal cut-offs. The area under the curve (AUC) was also calculated. The association of ferritin with C-reactive protein (CRP), white blood cells (WBC), ferrum and soluble transferrin receptor (STFR) was investigated *via* mixed linear regression models having ferritin as dependent variable. All reported *p*-values are two-tailed. Statistical significance was set at *p* < 0.05 and analyses were conducted using SPSS statistical software (version 22.0).

## Results

### Patient characteristics

**Figure 1:**
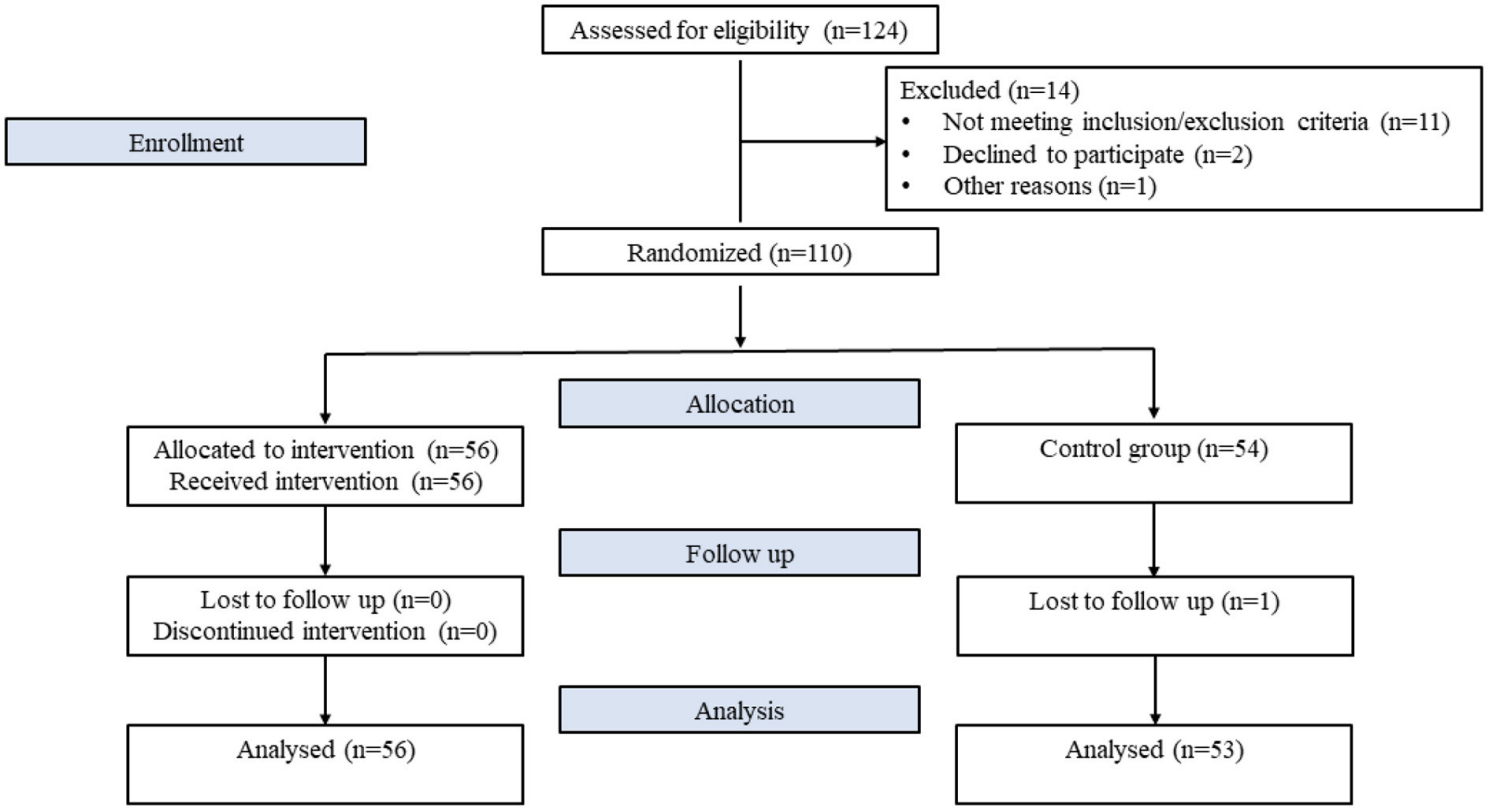
Study flowchart.

This study randomized 110 patients (124 patients were screened, but 14 patients were excluded, mainly due to inclusion/exclusion criteria) (Figure 1; Supplementary material, CONSORT 2010 Checklist). The overall baseline patient characteristics, as well as baseline characteristics split by the group type (dapagliflozin vs. control group), are presented in Table 1. Patients in the control group were older, had higher heart rate, urea, creatinine and uric acid compared to those on dapagliflozin. On the other hand, patients on dapagliflozin exhibited higher platelet, HbA1C and GFR values than the control group. The other baseline characteristics were not different between the 2 study groups. One patient from the control group was lost to follow up and there were no missing values.

**Table 1:** Baseline characteristics of the study population.

**Baseline characteristics**	**Overall** **(*N* = 110)**	**Dapagliflozin** **group (*N* = 56)**	**Control group** **(*N* = 54)**	***P*** **-value**
Age (years), mean ± SD	69.95 ± 9.33	68.11 ± 9.25	71.87 ± 9.10	**0.034**
Male sex, *N* (%)	90 (81.8)	47 (83.9)	43 (79.6)	0.559
Systolic blood pressure (mm Hg), median (IQR)	125.50 (28)	124.50 (26)	130 (31)	0.578
Diastolic blood pressure (mm Hg), mean ± SD	75.52 ± 11.88	75.96 ± 12.51	75.06 ± 11.29	0.690
Heart rate (bpm), median (IQR)	72 (14)	69 (11)	74.50 (17)	**0.014**
Left ventricular ejection fraction (%), median (IQR)	35 (10)	35 (15)	35 (10)	0.619
**New York Heart Association**, ***N*** **(%)**				
II, *N* (%)	53 (48.2)	24 (42.9)	29 (53.7)	0.255
III, *N* (%)	57 (51.8)	32 (57.1)	25 (46.3)	
Body weight (kg), median (IQR)	85 (25)	85 (27)	85.5 (25)	0.895
Body mass index (BMI), median (IQR)	27.7 (4.5)	27.6 (5.8)	27.9 (3.9)	0.654
**Comorbidities/Risk factors**				
Hypertension, *N* (%)	98 (89.1)	48 (85.7)	50 (92.6)	0.247
Atrial fibrillation, *N* (%)	36 (32.7)	15 (26.8)	21 (38.9)	0.176
Coronary artery disease, *N* (%)	103 (93.6)	51 (91.1)	52 (96.3)	0.438
Valvular disease, *N* (%)	11 (10)	5 (8.9)	6 (11.1)	0.703
Dyslipidemia, *N* (%)	104 (94.5)	52 (92.9)	52 (96.3)	0.679
Peripheral arterial disease, *N* (%)	25 (22.7)	12 (21.4)	13 (24.1)	0.741
Stroke, *N* (%)	17 (15.5)	7 (12.5)	10 (18.5)	0.383
Smoking, *N* (%)	36 (32.7)	21 (37.5)	15 (27.8)	0.277
**Laboratory blood values**				
Hematocrit (%), mean ± SD	39.98 ± 5.33	40.68 ± 5.90	39.25 ± 4.61	0.161
Hemoglobin (g/dl), mean ± SD	12.99 ± 1.93	13.16 ± 2.10	12.82 ± 1.71	0.352
MCV (fl), mean ± SD	85.88 ± 6.91	86.42 ± 6.87	85.32 ± 6.97	0.406
MCH (pg), mean ± SD	28.19 ± 2.86	28.31 ± 2.99	28.07 ± 2.73	0.667
MCHC (g/dl), median (IQR)	32.40 (1.6)	32.55 (1.4)	32.35 (1.8)	
White blood cells (cells/μl), mean ± SD	8,112.73 ± 1,858.629	8,240.36 ± 2,020.424	7,980.37 ± 1,683.248	0.466
Red blood cell distribution width (%), median (IQR)	14.60 (3.4)	14.60 (3.5)	14.60 (3.2)	0.974
Platelets (cells/μl), median (IQR)	219,500 (85,750)	238,000 (80,250)	210,500 (73,250)	**0.049**
Erythrocyte sedimentation rate (mm/h), median (IQR)	25.50 (28)	26 (27)	23 (30)	0.428
C-Reactive protein (mg/l), median (IQR)	6.25 (13.55)	6.03 (13.98)	7.98 (13.53)	0.421
Glucose (mg/dl), median (IQR)	153 (57)	146 (67)	156 (49)	0.724
HbA1C (%), median (IQR)	7.20 (1.4)	7.45 (1.3)	6.8 (1.3)	**<0.0001**
Urea (mg/dl), median (IQR)	48.50 (31)	45 (21)	55 (39)	**0.030**
Glomerular Filtration Rate (GFR)	68.8 (36.6)	72.0 (32.5)	61.1 (29.9)	**0.032**
**Chronic kidney disease stage**, ***N*** **(%)**				
G1	22 (20.0)	15 (26.8)	7 (13.0)	0.059
G2	48 (43.6)	26 (46.4)	22 (40.7)	
G3a	24 (21.8)	10 (17.9)	14 (25.9)	
G3b	11 (10.0)	5 (8.9)	6 (11.1)	
G4	5 (4.5)	0 (0.0)	5 (9.3)	
Creatinine (mg/dl), median (IQR)	1.10 (0.5)	1.03 (0.41)	1.16 (0.6)	**0.039**
SGOT (IU/l), median (IQR)	17.50 (10)	18 (10)	17 (10)	0.697
SGPT (IU/l), median (IQR)	28 (20)	27.50 (21)	28.50 (20)	0.592
Troponin (ng/ml), median (IQR)	0.04 (0.00)	0.04 (0.01)	0.04 (0.00)	0.492
K^+^ (mmol/l), mean ± SD	4.20 ± 0.46	4.25 ± 0.43	4.14 ± 0.49	0.222
Na^+^ (mmol/l), mean ± SD	139.73 ± 3.10	139.82 ± 2.91	139.63 ± 3.32	0.748
T3 (ng/ml), mean ± SD	0.92 ± 0.18	0.93 ± 0.17	0.91 ± 0.19	0.470
Ferrum (μg/dl), median (IQR)	60 (43)	62 (44)	60 (42)	0.879
Ferritin (ng/ml), median (IQR)	53.20 (87.6)	62.9 (99.3)	43.1 (76)	0.349
B12 (pg/ml), median (IQR)	228 (158)	241.5 (183)	217.5 (120)	0.274
Fil acid (ng/ml), median (IQR)	7.55 (3.9)	7.75 (4.2)	7.45 (3.8)	0.917
STFR (mg/l), median (IQR)	18.78 (11.14)	19.26 (10.63)	18.37 (11.63)	0.220
Brain natriuretic peptide (pg/ml), median (IQR)	199.95 (396)	183.95 (355)	211.15 (532.75)	0.462
Uric acid (mg/dl), median (IQR)	6.7 (2.5)	6 (2.3)	7.45 (2.8)	**0.004**
Erythropoietin (mIU/ml), median (IQR)	13.53 (12.06)	12.71 (12.59)	14.26 (11.78)	0.650
**Medical treatment**				
b-blocker, *N* (%)	102 (92.7)	52 (92.9)	50 (92.6)	1.000
ACE-i/ARB, *N* (%)	82 (74.5)	42 (75)	40 (74.1)	0.911
Sacubitril-Valsartan, *N* (%)	23 (20.9)	12 (21.4)	11 (20.4)	0.891
Mineralocorticoid antagonists, *N* (%)	71 (64.5)	37 (66.1)	34 (63)	0.733
Loop diuretics, *N* (%)	73 (66.4)	37 (66.1)	36 (66.7)	0.947
Antiplatelets, *N* (%)	69 (62.7)	38 (67.9)	31 (57.4)	0.257
Metformin, *N* (%)	97 (88.2)	50 (89.3)	47 (87)	0.715
Dipeptidyl peptidase-4-inhibitors, *N* (%)	50 (45.5)	22 (39.3)	28 (51.9)	0.250
Thiazolidinediones, *N* (%)	3 (2.7)	1 (1.8)	2 (3.7)	0.615
Sulfonylureas, *N* (%)	8 (7.3)	3 (5.4)	5 (9.3)	0.485
Insulin, *N* (%)	50 (45.5)	19 (33.9)	22 (40.7)	0.460

The bold values represent the p values that are < 0.05 (meaning that there is statistical significance).

### RDW longitudinal changes

**Figure 2:**
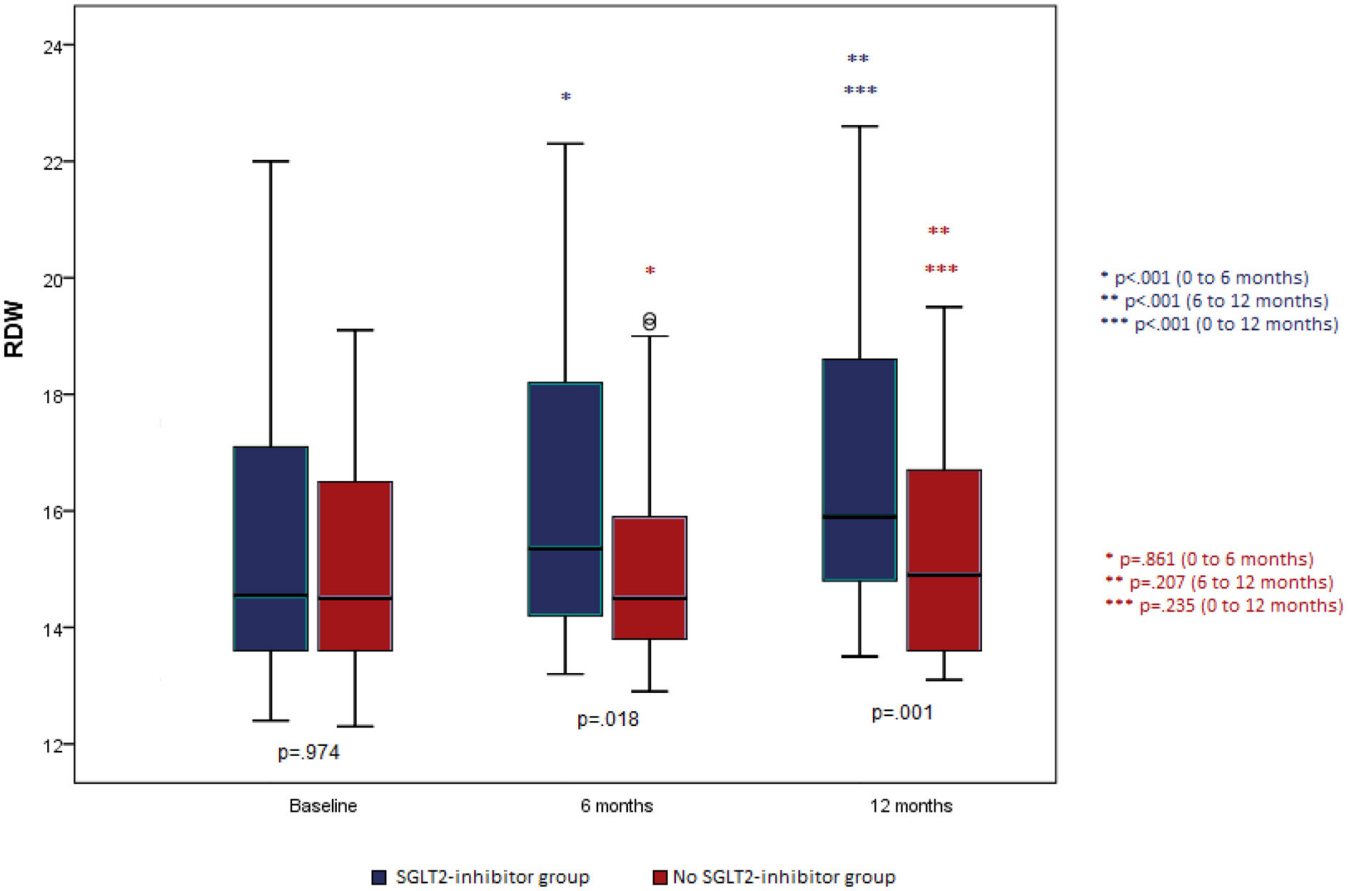
RDW change during the follow-up period for each group separately.

RDW was similar at baseline for both groups (*p* = 0.974). At 6 and at 12 months the SGLT-2i (dapagliflozin) group had significantly greater values compared to the control group (*p* = 0.018 and *p* = 0.001; Figure 2). Also, it was found that in the control group RDW was similar throughout the follow-up period, while in the SGLT-2i group there were significant increases between the consecutive measures as well as between baseline and last follow-up measurement (*p* < 0.001 for all time comparisons) (Figure 2).

After adjusting for all characteristics that differed significantly between the two groups at baseline ***(Model 1)***, RDW still increased significantly only in the SGLT-2i group over the follow-up period (Table 2). Age was positively correlated with RDW index. All other parameters included in the analysis were not significantly associated with RDW.

**Table 2:** Mixed linear regression results with RDW as dependent variable (after logarithmic transformation) and group, time, interaction term of group and time, and all characteristics differed significantly at baseline as independent variables (Model 1).

	**β+**	**SE++**	***P***
Time	0.0008	0.0010	0.381
**Group**			
**No SGLT-2i group (reference)**			
SGLT-2i group	0.0445	0.0249	0.074
Time*group	0.0061	0.0013	**<0.001**
Age	0.0044	0.0013	**0.001**
Heart rate	0.0001	0.0005	0.783
Platelets	0.0000	0.0000	0.496
HbA1C	−0.0076	0.0063	0.227
Urea	0.0002	0.0004	0.597
Creatinine	0.0580	0.0395	0.142
Uric acid	−0.0013	0.0041	0.752
GFR	0.0002	0.0005	0.635

+regression coefficient, ++standard error. The bold values represent the p values that are < 0.05 (meaning that there is statistical significance).

The analysis was repeated including parameters known from the literature that could be associated with RDW (Model 2, Table 3). It was found that RDW continued to increase significantly only in the SGLT-2i group over the follow-up period. Also, EPO was significantly and positively associated with RDW. On the other hand, ferritin and folic acid were significantly and negatively associated with RDW. Age, gender, hemoglobin, B12, uric acid, BMI, GFR, antiplatelets, and HbA1C were not significantly associated with RDW.

**Table 3:** Mixed linear regression results with RDW as dependent variable (after logarithmic transformation) and group, time, interaction term of group and time, age, EPO, Hb, Ferritin, B12, folic acid, uric acid, gender, BMI, GFR, antiplatelets, and HbA1C as independent variables (Model 2).

	**β+**	**SE++**	***P***
Time	0.0003	0.0010	0.721
**Group**			
No SGLT-2i group (reference)			
SGLT-2i group	0.0395	0.0217	0.069
Time*group	0.0056	0.0014	**<0.001**
Age	0.0024	0.0013	0.063
EPO	0.0007	0.0002	**0.001**
Hemoglobin	−0.0029	0.0049	0.544
Ferritin	−0.0003	0.0001	**<0.001**
B12	0.0001	0.0001	0.275
Folic acid	−0.0050	0.0019	**0.008**
Uric acid	0.0057	0.0039	0.140
**Gender**			
Women (reference)			
Men	0.0107	0.0278	0.701
BMI	−0.0008	0.0016	0.616
GFR	−0.0005	0.0003	0.055
**Antiplatelets**			
No (reference)			
Yes	−0.0311	0.0222	0.162
HbA1C	−0.0024	0.0060	0.691

+regression coefficient, ++standard error. The bold values represent the p values that are < 0.05 (meaning that there is statistical significance).

### Outcomes

Altough, the percentage of 1-year death or HF rehospitalization was numerically lower in the SGLT-2i group (8.9% for the dapagliflozin group vs. 16.7% for the control group), there was no significant difference between the two groups (*p* = 0.223; Supplementary Table 1).

### Baseline RDW and outcome

RDW was significantly and independently associated with 1-year death or HF rehospitalization, after adjusting for age, group, gender, smoking and BMI at baseline. More specifically, greater RDW values at baseline were significantly associated with greater probability of death or rehospitalization, i.e., worse prognosis. All other independent factors were not found to be significantly associated with prognosis (Table 4).

**Table 4:** Multiple logistic regression results with 1-year death or HF rehospitalization as dependent variable.

	**OR (95% CI)^+^**	***P***
**RDW (at baseline)**	**1.40 (1.07–1.83)**	**0.014**
**Group**		
No SGLT-2i group (reference)		
SGLT-2i group	0.42 (0.12–1.56)	0.196
**Gender**		
Women (reference)		
Men	0.54 (0.10–2.89)	0.468
Age	1.04 (0.96–1.13)	0.292
**Smoking**		
No (reference)		
Yes	0.89 (0.22–3.64)	0.870
BMI (at baseline)	1.00 (0.90–1.11)	0.989

+Odds ratio (95% Confidence Interval). The bold values represent the p values that are < 0.05 (meaning that there is statistical significance).

The prognostic value of RDW is listed in the Supplementary Figure 1. Baseline RDW was of prognostic significance regarding the combined endpoint of 1-year death/rehospitalization in both groups [AUC 0.82, 95% CI (0.68–0.96), *p* = 0.019 in the dapagliflozin group and AUC 0.72, 95% CI (0.56–0.87), *p* = 0.042 in the control group]. No significant difference was observed between the aforementioned AUCs (*p* = 0.349). Optimal RDW cut-off for the dapagliflozin group was 15.7%, with 80% sensitivity and 70.6% specificity. For the control group, optimal RDW cut-off value was 14.8%, with 77.8% sensitivity and 57.8% specificity.

### Association of ferritin with markers of iron overload and inflammation

Greater WBC and ferrum values were significantly associated with greater ferritin values. On the contrary, soluble transferrin receptor (STFR) was significantly negatively associated with ferritin. CRP was not significantly associated with ferritin (Supplementary Table 2).

### Adverse events

No serious adverse events (diabetic ketoacidosis, symptoms of volume depletion, renal events, major hypoglycemia, fracture, lower limb amputations, Fournier's gangrene), and adverse events leading to discontinuation of the study drug were reported. Urinary tract infection was reported in two patients (one in the dapagliflozin group and 1 in the control group).

## Discussion

The present study demonstrated a correlation between the SGLT-2i (dapagliflozin) use, and an increase in the rates of RDW in patients with HF and DM both after 6 and after 12 months against those not on SGLT-2i (control group). It was equally noted that the increase in RDW was positively correlated with factors such as EPO, while negatively correlated with ferritin and folic acid. Lastly, baseline RDW proved to be independently associated with outcomes (death or HF rehospitalization) at 1-year, in both groups.

### Potential mechanisms of RDW increase

HF and DM often coexist and share common pathophysiological routes such as oxidative stress, inflammation and the disruption in normal hematopoiesis, conditions which can lead patients in developing anemia of chronic disease (8). EPO is a hormone produced by the kidneys and secreted in situations of hypoxia; its principal function is to induce hematopoiesis (25, 26). The increase in hematopoiesis which happens through erythropoietin results in an increase of the size of the circulating RBCs and consequently in an increase of the rate of RDW (anisocytosis) (27).

SGLT-2i are antidiabetic medications with pleiotropic effects in multiple organs and proven cardiovascular benefits, especially in patients with HF and DM (28). One of their favorable actions is the increase in the levels of Ht although the exact mechanism through which this happens is unknown (29). A sub-analysis of EMPA-HEART CardioLink-6 (Effects of Empagliflozin on Cardiac Structure in Patients With Type 2 Diabetes) randomized clinical trial, including 82 patients with DM and coronary artery disease showed that the administration of empagliflozin was associated with an increase in the EPO levels, a change in the morphology of RBCs (increase in RDW), a decrease in ferritin reserves and an increase in Ht concluding that SGLT-2i induce hematopoiesis through the increase in the secretion of EPO (15). Another randomized study investigated the effect of dapagliflozin on the levels of Ht and hepcidin (a suppressive hormone for hemopoiesis which increases in pre-inflammatory conditions such as DM or HF) in 52 patients with type 2 DM (30). Patients who were on dapagliflozin presented an increase in Ht and EPO, but a decrease in the rate of hepcidin and ferritin. The investigators concluded that dapagliflozin induces hematopoiesis through the mobilization of iron reserves, increase in EPO and suppression of hepcidin (30). The present study demonstrated that dapagliflozin administration increases RDW values in patients with HF and DM and that this increase is associated with the EPO rates. Taking into consideration that the induction of hemopoiesis through the increase of endogenous EPO leads to anisocytosis (and by extension to an increase in RDW), it is possible that these observations suggest the stimulation of hemopoiesis from dapagliflozin in patients with HF and DM. This supposition is reinforced by the fact that a negative correlation between RDW and ferritin as well as folic acid was noted, which suggests a potential mobilization of the reserves of endogenous iron and folic acid for the induction of hemopoiesis as necessary ingredients in the composition of RBCs. Furthermore, the present study revealed a positive association between ferritin values and ferrum as well as WBC (markers of iron and inflammation, respectively), and a negative association between ferritin and STFR (marker of erythropoiesis). Potential mechanisms through which a stimulation of hemopoiesis through SGLT-2i can occur have not yet been clarified (30–32).

Another parameter which has been correlated with higher RDW rates is increased age. In a retrospective study of 1907 healthy subjects, RDW rates increased according to the age group (*p* < 0.001) while the average rate of RDW was 11% higher in persons aged over 60 years compared to persons aged under 60 years (14.6 vs. 13.2%; *p* < 0.001) (33). Similar findings were reported in a study utilizing data from 8,089 unique individuals, reporting an RDW increase of 6% from the youngest to oldest age class (34). In the present study, RDW rates were positively correlated with aging in Model 1. Taking into consideration that increase in age parallels an increase in inflammation, oxidative stress as well as deficiencies in basic ingredients for the maturing RBCs such as folic acid, vitamin B12 and iron, it can be suggested that all of the above pathologies lead to disorders in normal hemopoiesis and by extension to presenting anisocytosis (35, 36).

Based on all the above, it could be proposed that the administration of SGLT-2i appears to result in the induction of hemopoiesis in patients with HF and DM (Supplementary Figures 2, 3) resulting in RDW increase. Notably in the present study, the abovementioned cardioprotective mechanism of SGLT-2i didn't result in better outcome in the dapagliflozin group vs. the control group. However, this may be due to the small number of adverse events observed in both groups.

RDW is an important marker of prognosis in HF patients (37, 38). In the present study, the administration of SGLT-2i (dapagliflozin) was associated with the increase of RDW over time. Having in mind, that the cardioprotective actions of SGLT-2i in HF patients are established (39–42), this beneficial effect on prognosis might be mediated by mechanistic actions not comprehensively investigated in the present study, such as the reduction in oxidative stress involving the xanthine oxidase pathway (43, 44). Oxidative stress has also been suggested to increase RDW rates (45, 46). One of the endogenous sources of reactive oxygen species (ROS) is the enzyme xanthine oxidase which catalyzes hypoxanthine to xanthine and xanthine to uric acid. Oxidative stress has an important role in the onset and development of HF and DM (47, 48) while studies have highlighted the prognostic value of uric acid as a potential indicator of increased oxidative stress in both conditions (49–52). However, uric acid may be caused not only by oxidative stress but also by decreased uric acid excretion from the kidneys and high purine diets (53). The present study was not able to demonstrate any association between uric acid and RDW.

### RDW as a prognostic indicator

The value of RDW as a prognostic indicator in patients with HF and DM has been previously reported (11, 54). The present study adds to the current knowledge by showing that the prognostic value of baseline RDW in patients with HF and Type 2 DM was significant both in patients who started receiving SGLT-2i (dapagliflozin) or not. Therefore, in the era of novel life saving therapies (SGLT-2i) in HF, baseline RDW remains a simple, inexpensive and reliable prognostic marker.

It is well-known from the literature, that a high RDW value in patients with DM and HF is likely a consequence of a chronic inflammatory state involving a reduced use of iron reserves and reduced production of erythropoietin and correlates negatively with prognosis (11, 54). However, the pathophysiological significance of an increased RDW at follow-up in patients treated with SGLT-2i (dapagliflozin) could be different, and likely linked to a greater use of iron reserves and increase in erythropoiesis as demonstrated in the present work (Supplementary Table 3). Large, prospective clinical studies are urgently needed.

### Study limitations

The sample size was not large (110 patients), however each patient completing the follow-up had 3 measurements (baseline, 6 and 12 months). Another limitation was the lack of double blind and placebo group design, although patients were treated on top of optimized medical therapy. Consequently, the results of this study should be evaluated with caution and used as a basis for larger studies. Furthermore, the SGLT-2i used in the current study was dapagliflozin and therefore the current observations may not apply to other SGLT-2i. However, the possibility that those mechanisms reflect a “class effect” can't be excluded.

## Conclusion

RDW, a simple parameter of a blood count which indicates anisocytosis, has been found in this study to increase in patients with HF and DM who received SGLT-2i (dapagliflozin). The increased RDW rates in these patients may be due to the induction of hemopoiesis from dapagliflozin. RDW was independently associated with 1-year death or HF rehospitalization, in patients with DM and HF.

## Data availability statement

The raw data supporting the conclusions of this article will be made available by the authors, without undue reservation.

## Ethics statement

The studies involving human participants were reviewed and approved by Institutional Review Committee of the Konstantopoulio General Hospital (email: grammateia.ep.symvouliou@konstantopouleio.gr). The patients/participants provided their written informed consent to participate in this study.

## Author contributions

NK and EM collected the data. AX, GG, II, SP, FT, and JS conceived and designed the study. SP, II, and JS were responsible for the project administration. II, FT, and JS were responsible for the supervision of the study. NK, AX, and JS wrote the main manuscript text. GG, SS, and EM prepared figures. NK (biostatistician) performed the statistical analysis. II, SP, and FT revised the manuscript critically for important intellectual content. All authors reviewed the manuscript, discussed the results, and commented on the manuscript.

## Conflict of interest

The authors declare that the research was conducted in the absence of any commercial or financial relationships that could be construed as a potential conflict of interest.

## Publisher's note

All claims expressed in this article are solely those of the authors and do not necessarily represent those of their affiliated organizations, or those of the publisher, the editors and the reviewers. Any product that may be evaluated in this article, or claim that may be made by its manufacturer, is not guaranteed or endorsed by the publisher.
