# Supplementary material for: The effect of SGLT-2i administration on red blood cell distribution width in patients with heart failure and type 2 diabetes mellitus: A randomized study
Source: Front Cardiovasc Med. 2022 Sep 29;9:984092. doi: 10.3389/fcvm.2022.984092 (PMC9557218; doi:10.3389/fcvm.2022.984092)
Supplement: Supplementary file 2 [file Data_Sheet_2.DOCX]

**Supplementary Material**

**Suppl. Figure 1.** Receiver operating characteristic curves (ROC) to assess the prognostic value of baseline RDW (as a continuous variable) for the combined endpoint of death/HF rehospitalization at 12 months A) in the SGLT-2i (dapagliflozin) group and B) the control group.


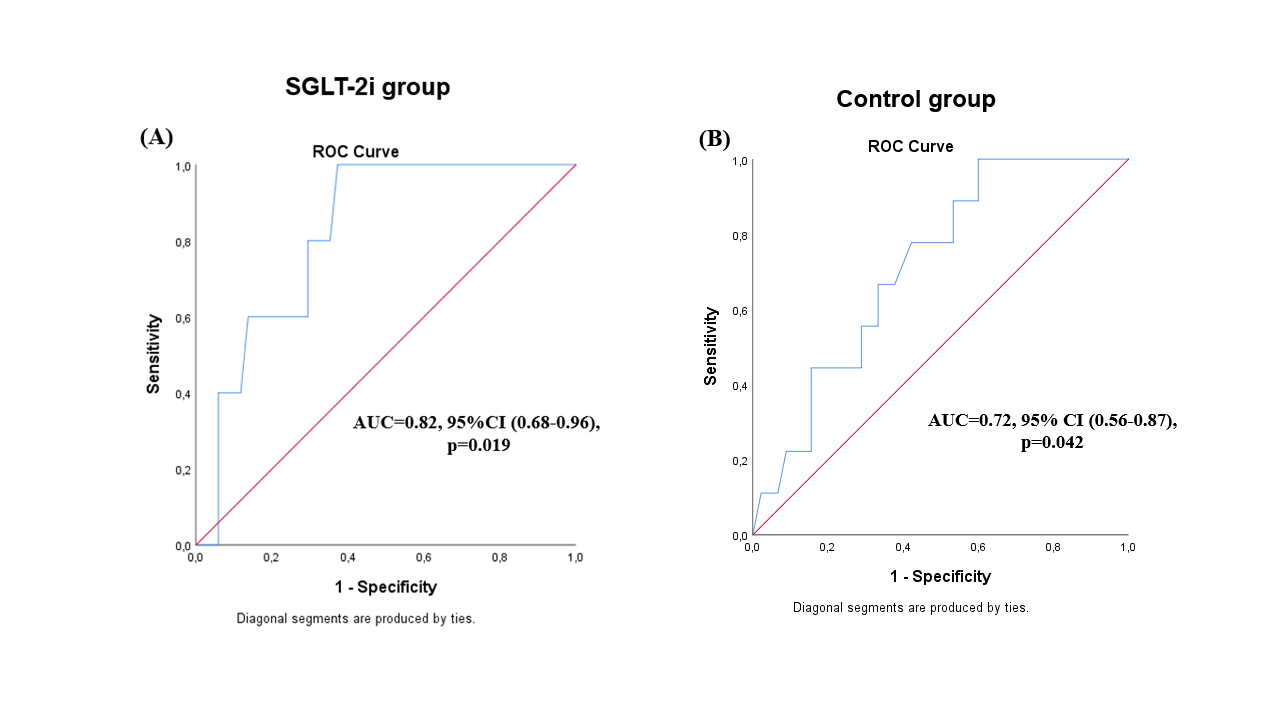


No significant difference was observed between the aforementioned AUCs (p=0.349).

**Suppl. Figure 2.** Hemoglobin longitudinal changes in the dapagliflozin and control group.


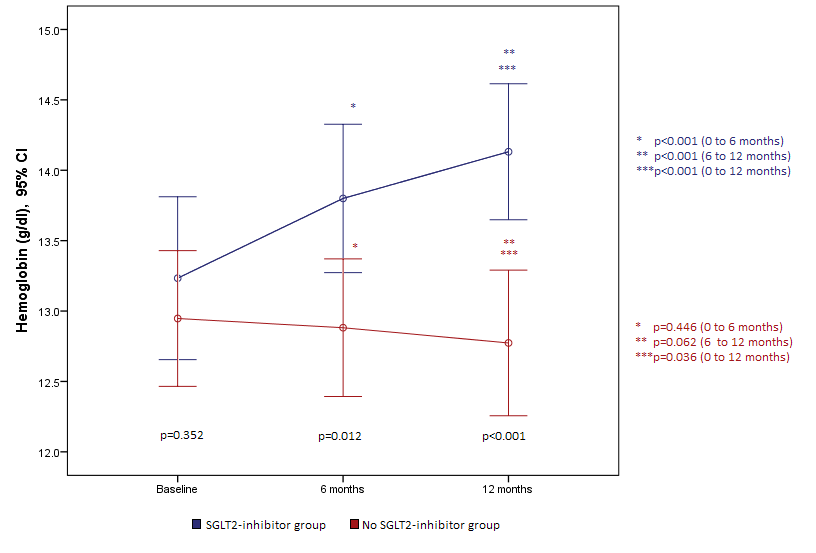


**Suppl. Figure 3.** Hematocrit longitudinal changes in the dapagliflozin and control group.


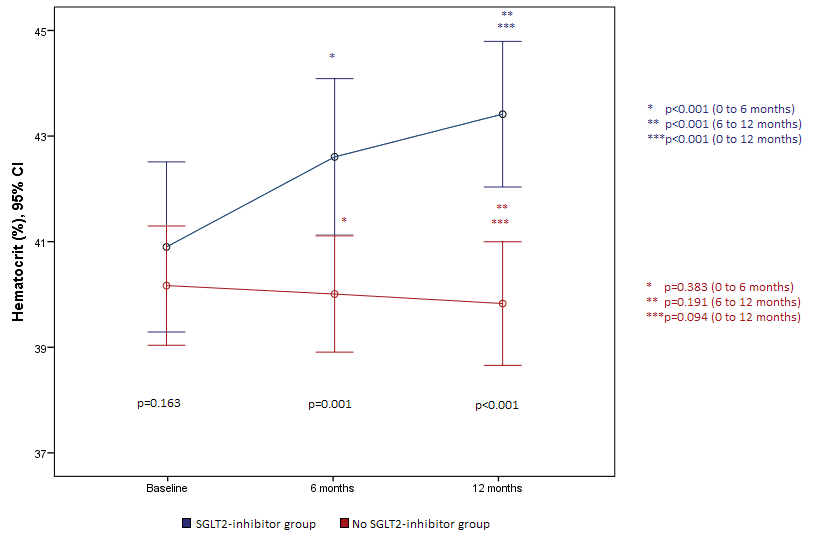


**Suppl. Table 1.** Percentages of 1-year death or rehospitalization in the two groups (Dapagliflozin vs control group)

|  | Death or Rehospitalization | | | | | P Pearson's x^2^ test |
| --- | --- | --- | --- | --- | --- | --- |
|  | No | | | Yes | |  |
|  | N | % | N | | % |  |
| Dapagliflozin group | 51 | 91,1 | 5 | | 8,9 | 0,223 |
| Control group | 45 | 83,3 | 9 | | 16,7 |  |

**Suppl. Table 2.** Univariate mixed linear regression results with ferritin as dependent variable.

| Independent variables | β+ | SE++ | P |
| --- | --- | --- | --- |
| CRP | 0.37 | 0.34 | 0.275 |
| WBC | 0.004 | 0.002 | 0.047 |
| Fe | 1.23 | 0.17 | <0.001 |
| STFR | -2.40 | 0.56 | <0.001 |

+regression coefficient; ++Standard Error

**Suppl. Table 3.** Correlation between RDW and hematocrit (Ht)/ hemoglobin (Hb) changes (from baseline to 12 months) in the SGLT-2i group (dapagliflozin)

| Variable | Spearman’s rho | P-value |
| --- | --- | --- |
| RDW-Ht | 0.30 | 0.033 |
| RDW-Hb | 0.24 | 0.094 |

In the dapagliflozin group it was found that greater increase in hematocrit was significantly associated with greater increase in RDW values, rho=0.30; p=0.033. Also, in the aforementioned group, it was found that greater increase in hemoglobin tended to be associated with greater increase in RDW values, rho=0.24; p=0.094. RDW was not changed significantly in the control group, thus no further correlation analysis was conducted in this group.
